# Differentiated stimulating effects of activated carbon on methanogenic degradation of acetate, propionate and butyrate

**DOI:** 10.1016/j.wasman.2018.03.037

**Published:** 2018-06

**Authors:** Suyun Xu, Runqi Han, Yuchen Zhang, Chuanqiu He, Hongbo Liu

**Affiliations:** Department of Environment & Low-Carbon Science, School of Environment and Architecture, University of Shanghai for Science and Technology, Shanghai 200093, China

**Keywords:** Granular activated carbon, Interspecies electron transfer, Methanogenesis, Syntrophic oxidization, Volatile fatty acids

## Abstract

Granular activated carbon (GAC) could promote methane production from organic wastes, but a wide range of dosages has been reported. In present study, different GAC dosages of 0, 0.5, 5 and 25 g/L were supplemented into anaerobic digesters and the methanogenic degradation kinetics of acetate, propionate and butyrate were characterized, respectively. At high organic load of 5 g/L, the degradation rates of propionate and butyrate increased by 1.5–4.7 and 2.5–7.0 times at varied GAC dosages. The methane production rates (*R_max_*) from propionate and butyrate were significantly elevated when increasing GAC dosage up to 5 g/L. However, only a minor increment was found for acetate degradation either at 1 g/L or 5 g/L. The stimulatory mechanism of GAC for accelerated syntrophic degradation of propionate and butyrate can be primarily attributed to the triggering effect on acetogenesis, as evidenced by the enrichment of syntrophic bacteria e.g. *Thermovirga, Synergistaceae,* and *Syntrophomonas* etc.

## Highlights

• Anaerobic digesters with varied GAC dosages were compared at low and high OLRs.
• Methanogenic degradation of acetate was almost unaffected by GAC addition.
• GAC dose-dependent enhancing effect was found for syntrophic HPr/HBu degradation.
• GAC stimulated the enrichment of syntrophic bacteria*.*

## Graphical abstract

**Figure:**
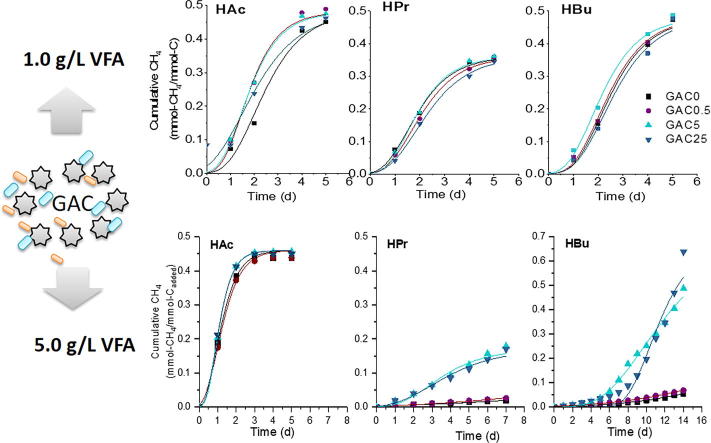

## 1 Introduction

Biomethane production through anaerobic digestion is one of the most successful strategies utilizing bio-energy worldwide (Ferguson et al., 2016, Xiao et al., 2013). In general, anaerobic methanogenesis is carried out by several groups of microorganisms involved in the hydrolysis, acidogenesis, acetogenesis and methanogenesis processes. Fermentative bacteria and acetogens produce volatile fatty acids (VFAs) and other intermediates, such as lactate, ethanol and butanol and, etc., from the degradation of complex macromolecules (Karthikeyan and Visvanathan, 2013, Lee et al., 2016). Methanogens utilize simple organic substrates, such as acetate, CO_2_/H_2_, methanol, and formate to generate methane (Mcinerney et al., 1981, Pan et al., 2016, Stuckey and David, 1999).

As VFAs other than acetate can’t be directly used by methanogens and therefore propionic and butyric acids are mostly found in the effluent from digester with high loads (Li et al., 2017, Viggi et al., 2014). In fact, the oxidation of propionate and butyrate are highly endergonic under standard conditions and occurs only if methanogens keep the concentrations of these intermediate products low (Müller et al., 2010). Propionate and butyrate are firstly converted to acetate and CO_2_/H_2_ by acetogens, and then they are utilized by aceticlastic- and hydrogenotrophic- methanogens. Syntrophic interspecies H_2_ transfer is essential to make the reaction energetically favorable (Müller et al., 2010, Schink, 1997).

There are considerable studies aiming to strengthen the syntrophic metabolism within methanogenic conditions by supplementing conductive iron oxides, such as magnetite and Fe^0^ (De Vrieze et al., 2016, Yamada et al., 2015) or conductive carbon materials, such as activated carbon (Liu et al., 2012, Xu et al., 2015), biochar (Luo et al., 2015), carbon cloth and graphite (Dang et al., 2017, Lee et al., 2016, Mumme et al., 2014, Zhao et al., 2015) etc. The stimulated methane production in reactors with conductive materials might be attributed to the promotion of direct interspecies electron transfer (DIET) (Li et al., 2017, Liu et al., 2012, Rotaru et al., 2014a, Rotaru et al., 2014b). One potential reason for this is that the availability of non-biological conductive materials may save cells energy because they do not need to produce as extensive extracellular biological electrical connections, such as electrically conductive pili and c-type cytochromes (Zhao et al., 2015).

Carbon materials could also provide high specific area for the effective immobilization of syntrophic microorganisms (Li et al., 2017, Luo et al., 2015, Kindzierski et al., 1992). Zhao et al. (2016) found that the abundance of *Geobacter* species, such as *G. sulfurreducens* and *G. lovleyi* increased in the propionate- and butyrate-fed reactors, accounting for 20% of the community attached to biochar, meanwhile *Syntrophomonas* and *Smithella* species declined. Nevertheless, Dang et al. (2017) reported that granular activated carbon (GAC) seemed to significantly increase the abundance of syntrophic bacteria such as *Syntrophomonas, Symbiobacterium* and *Desulfotomaculum* species, whereas *Geobacter* were not enriched in any of the OFMSW reactors supplemented with GAC. The distinct results might either attributed to the different inoculums or different carbon sources, e.g. single/mixed VFAs, ethanol or complex organic matters (Kato et al., 2012, Wang et al., 2016). Thus further investigations are in demand to understand the syntrophic communities for propionate and butyrate in digester with carbon materials.

Furthermore, it is noted that only simple comparison between AC treated group and blank group has been reported in most studies, and the supplementing dosage of AC varied widely (e.g. from 0.005 to 50 g/L), as shown in Table S1. Nevertheless, Chen et al. (2014) reported that the metabolism rates of ethanol in methanogenic reactor increased when the amount of carbon cloth was doubled from 10 g/L to 20 g/L, but without further interpretation. Therefore, it is also necessary to clarify whether there is a dose-dependent effect and provide a quantitative basis for related practices.

Based on the above rationale, this study has investigated the degradation kinetics of acetate, propionate and butyrate, separately in methanogenic digesters supplemented with a series of GAC dosages (i.e. 0.5–25 g/L). Meanwhile, two different organic loads of substrate, i.e. 1 g/L and 5 g/L were compared. The rates of VFAs’ degradation and methane generation were evaluated by using first-order kinetics and Modified Gompertz model. The high throughput technique was used for 16s rDNA sequencing to detect the microbial community structure, and the alternation of syntrophic VFAs degrading bacteria and methanogens due to GAC addition was discussed in this study.

## 2 Methods

### 2.1 Preparation of sludge inoculum and experimental design

Inoculum sludge taken from Quyang Sewage Treatment Plant (Shanghai, China) was pre-cultured in a laboratory scale anaerobic digester. And then the sludge was transferred to three reactors fed with different VFAs, i.e. acetate, propionate and butyrate, respectively, to enrich the specific fatty acids degradation bacteria. After several sequential batches of cultivation, 1 g/L of each VFA species could almost be degraded after 5–7 days. The cultivation temperature was maintained at 35 ± 2 °C.

During experiments, the determined volume of enriched sludge was put into 500 mL serum bottle with 400 mL of digestate liquid to make a final concentration of total volatile suspended solid (TVSS) at 1 g/L. Different dosages of GAC was supplemented to serum bottles i.e. 0, 0.5, 5 and 25 g/L, respectively, which were recorded as GAC0, GAC0.5, GAC5 and GAC25. GAC was purchased from Sinopharm Chemical Reagent CO. LTd. 20–40 mesh GAC was obtained by shive, which apparent density and specific surface area was 430 ± 30 g/L and 875–1185 m^2^/g, respectively. Sequentially, the conversion rates of acetate, propionate and butyrate into methane with specific enriched cultures were evaluated in batch studies at the concentration of 1 g/L and 5 g/L. Data was collected after two batches of pre-culture, and each test was carried out in triplicate. The temperature of reactors was maintained at 35 ± 2 °C with an incubator shaker (DKY-II, Shanghai Duke Auto Co., China).

The substrate formula: specific carbon source (e.g. acetate, propionate and butyrate), the corresponding qualities of NH_4_Cl and KH_2_PO_4_ were added to the reactors according to the C: N: P = 100:5:1. Additionally, 2 mL/L of the trace element solution was added (El-Mamouni et al., 1995) and the pH was adjusted to 7.2 with HCl and NaOH solutions. Finally, all reactors were flushed with nitrogen gas for more than 10 min before startup.

### 2.2 Physiochemical analyses

The volume of methane generation was automatically measured by AMPTS II (Bioprocess, Sweden) equipped with a gas flowmeter. The liquid of each reactor was sampled and analyzed to monitor the variations of total organic carbon (TOC) and VFAs. After filtrated by 0.45 μm filter membrane, the concentration of VFAs was analyzed by high performance liquid chromatography (Waters 2695/2489, USA) equipped with refractive index detector. The TOC was analyzed by Total Carbon/Total Nitrogen analyzer (Multi N/C 3100, Jena Co., Germany).

### 2.3 Microbial community analyses

The sludge samples were collected from GAC0 and GAC5 reactors at the end of experiment. The total DNAs of all samples were extracted using the Power Soil^TM^ DNA isolation kit (Mo-Bio Laboratories Inc., CA). Labels of “HAc0”, “HPr0”, “HBu0” stand for the sludge samples taken from GAC0 with respective substrate, and “HAc1”, “HPr1”, “HBu1” stand for the samples taken from GAC5. The microbial community of samples was analyzed by using high-throughput pyrosequencing on an Illumina platform (Illumina Miseq PE300). Amplicon libraries were constructed for pyrosequencing using bacterial primers 515F (50-GTG CCA GCM GCC GCG GTA A-30) and 806R (50-GGA CTA CHVGGG TWT CTA AT-30) for the V4–V5 region of the microbial 16SrRNA gene (Xu et al., 2015). Sequencing data has been deposited into public database NCBI, and the accession number is SRP134710 (https://www.ncbi.nlm.nih.gov/sra/SRP134710).

### 2.4 Data analysis

Modified Gompertz model (Eq. (1)) was fitted to the experimentally observed curve of cumulative CH_4_ production (Lü et al., 2013). The variations of VFAs were fitted with first-order kinetics (Eq. (2)).

(1) $$M_{{\mathrm{CH}}_{4}}(t)=P_{{\mathrm{CH}}_{4}}\times \exp\left\{-\exp\left[\frac{R_{{\mathrm{CH}}_{4}}\times e}{P_{{\mathrm{CH}}_{4}}}\times (\lambda_{{\mathrm{CH}}_{4}}-t)+1\right]\right\}$$

(2) $$\ln\frac{C_{0}}{C(t)}=\mathrm{kt}$$

Where, in Eq. (1), M(t), P, R and λ is cumulative production (mmol-C/mmol-C_added_) at time t, ultimate methane yield (mmol-C/mmol-C_added_) at the end of the incubation, maximum production rate (mmol-C/mmol-C_added_/d) and lag phase (d), respectively for CH_4_ and CO_2_ production; e is 2.71828. In Eq. (2), *C_0_* and *C*(*t*) is the initial concentration of particular substrate and the concentration at time t; *k* is the first order degradation constant.

## 3 Results and discussion

### 3.1 Profile of VFAs degradation and methane generation at low strength

**Fig. 1:**
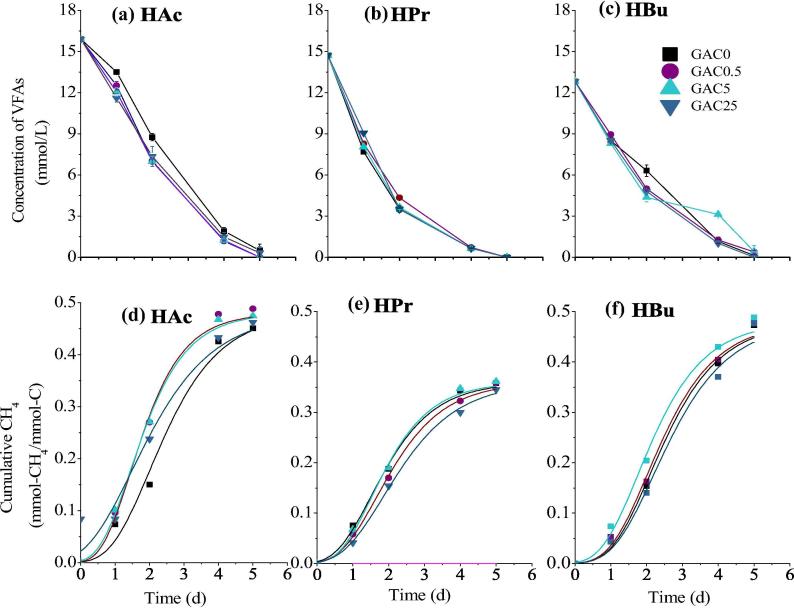
Degradation profiles of 1 g/L VFA (a–c) and corresponding methane production (e–h) with different dosages of GAC.

**Fig. 2:**
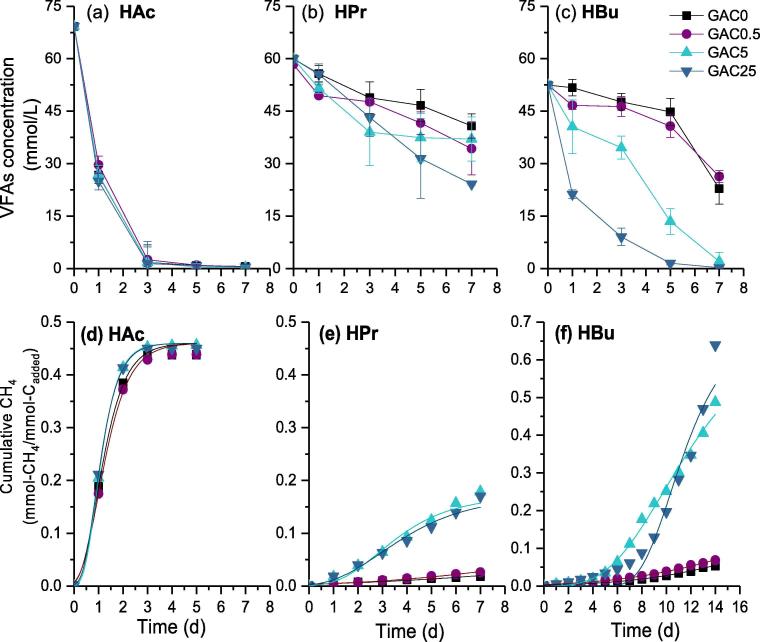
Degradation profiles of 5 g/L of VFA (a–c) and corresponding methane production (e-h) with different dosages of GAC.

Syntrophic interaction is essential to overcome the thermodynamic barriers in the anaerobic oxidation of fermentation intermediates especially propionate and butyrate (Hattori, 2008). In present study, we examined the methanogenic degradation of HAc, HPr and HBu, respectively with the supplementation of GAC at different organic loads, i.e. 1 g/L and 5 g/L, which profiles of cumulative methane production and VFAs declination are presented in Figs. 1 and 2.

**Fig. 3:**
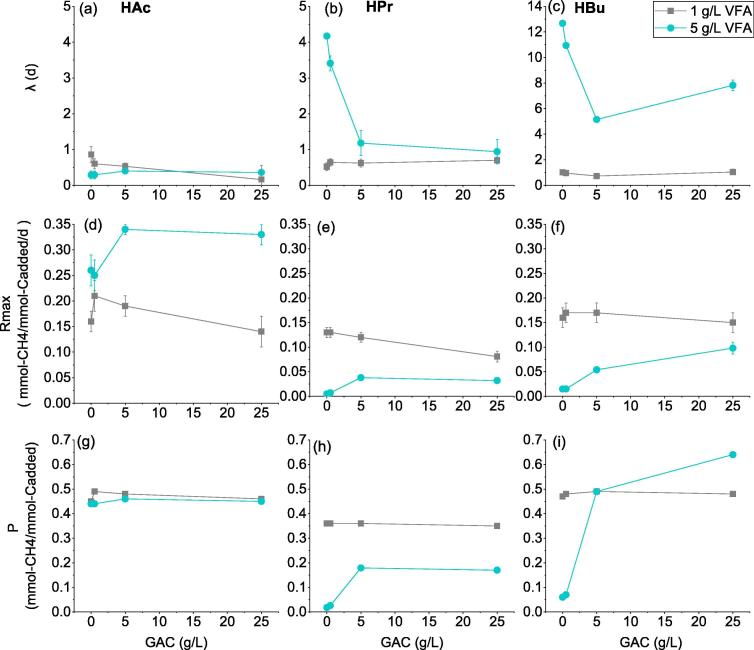
The calculated kinetic values of lag phase λ (a–c), maximum production rate *R_max_* (d–f) and ultimate methane yield *P_CH4_*, (g–i) from Modified Gompertz model.

With the initial concentration of 1 g/L, three species of VFAs i.e. HAc, HPr and HBu were rapidly degraded, which almost vanished after 5 days. Similarly, the lag phase of methane generation could be neglected. It indicates that the enriched microbial consortia have strong metabolic ability for the specific substrate, i.e. acetate, propionate and butyrate. Furthermore, there is no obvious difference among the reactors with the same substrate and different dosage of GAC as shown in Fig. 1. The calculated kinetic values of ultimate methane yield (*P_CH4_*), maximum production rate (*R_max_*), and lag phase (λ) from Modified Gompertz model were presented in Fig. 3. The *P_CH4_* from acetate was 0.45–0.49 mmol-CH_4_/mmol-C_added_, which was close to the theoretical value of 0.5 mmol-CH_4_/mmol-C_added_. The average *R_max_* of HAc was 0.16 mmol-CH_4_/mmol-C_added_/d, which value was slightly higher than the previous study, i.e. 0.107–0.143 mmol-CH_4_/mmol-C_added_/d (Lü et al., 2013). Meanwhile *P_CH4_* from propionate and butyrate were slightly lower than their theoretical values, i.e. 0.48 and 0.68 mmol-CH_4_/mmol-C_added_. Thus the strengthening effect of GAC at low strength was not prominent.

### 3.2 Profile of VFAs degradation and methane generation at high strength

With the initial concentration of 5 g/L, acetate-acclimated culture still showed high metabolic activity and the supplementation of GAC almost did not affect the methane production rate. The ultimate methane yielding *P****_CH_****_4_* from acetate was around 0.45 mmol-CH_4_/mmol-C_added_, which was close to the value obtained in low strength of acetate (e.g. 1 g/L).

However, the methanogenic conversion of propionate and butyrate were obviously inhibited when increasing the substrate concentration from 1 g/L to 5 g/L. The lag-phase time of propionate and butyrate reactors was 4.2 days and 12.7 days for GAC0. It is like previous studies that methanogenesis was vulnerable to high concentration of VFAs, mainly attributing to the inhibition of growth and metabolism of methanogens by undissociated VFAs (Pavlostathis and Giraldo-Gomez, 1991). Nevertheless, the addition of GAC in present study was found to accelerate the metabolism of propionate and butyrate significantly. As shown in Fig. 2, when increasing GAC concentration from 0.5 g/L to 25 g/L, the lag-phase period reduced from 3.4 d to 0.9 d for propionate-fed reactors, and from 12.7 d to 7.8 d for butyrate-fed reactors.

The degradation kinetics (*k*) of each VFAs species are calculated and presented in Table 1. Basically, the R^2^ is high, only the R^2^ of HBu (5.0 g/L) was lower than others especially at low GAC dosage. The reason could be attributable to the inhibition effect, leading to the deviation from sigmoidal function. With 5 g/L of propionate as substrate, about 1.5 times of increment was found for the *k* value in digesters, i.e. from 0.0022 to 0.0056 h^−1^ when increasing GAC dosage from 0 to 25 g/L. The stimulating effect on degradation of butyrate was more significant, which kinetics value was enhanced by 7.1 times i.e. from 0.0043 to 0.0306 h^−1^ when GAC dosage increased from 0 to 25 g/L. The above results clearly indicated that the supplementation of GAC could accelerate methanogenesis from propionate and butyrate in a dose-dependent manner. Li et al. (2015) also reported about the stimulating effect of nano-Fe_3_O_4_ might be limited by the supplementing concentration. It should also be noted that although the degradation rates of propionate and butyrate were significantly increased by supplementing GAC, their rates were still lower than that of acetate in the range of 0.0386–0.0431 h^−1^. These results further confirmed the difficulty in conversion of propionate and butyrate and the importance to further explore the promotion mechanism.

**Table 1:** First-order kinetics for the consumption rate of propionate and butyrate.

	GAC dosage	HAc		HPr		HBu	
VFA		*k* (h^−1^)	*R^2^*	*k* (h^−1^)	*R^2^*	*k* (h^−1^)	*R^2^*
	0 g/L	0.0386 ± 0.0065	0.92	0.0022 ± 0.0002	0.97	0.0043 ± 0.0015	0.83
5.0	0.5 g/L	0.0371 ± 0.0049	0.95	0.0033 ± 0.0004	0.92	0.0036 ± 0.0009	0.77
g/L	5 g/L	0.0431 ± 0.0046	0.97	0.0029 ± 0.0041	0.75	0.0180 ± 0.0041	0.82
	25 g/L	0.0393 ± 0.0078	0.90	0.0056 ± 0.0004	0.99	0.0306 ± 0.0021	0.98
	0 g/L	0.0228 ± 0.0042	0.91	0.0328 ± 0.0013	0.99	0.0251 ± 0.0040	0.99
1.0	0.5 g/L	0.0279 ± 0.0044	0.93	0.0319 ± 0.0025	0.98	0.0247 ± 0.0022	0.98
g/L	5 g/L	0.0274 ± 0.0041	0.94	0.0329 ± 0.0017	0.99	0.0248 ± 0.0031	0.99
	25 g/L	0.0252 ± 0.0036	0.94	0.0333 ± 0.0023	0.99	0.0268 ± 0.0025	0.99

### 3.3 Variations of intermediate products

**Fig. 4:**
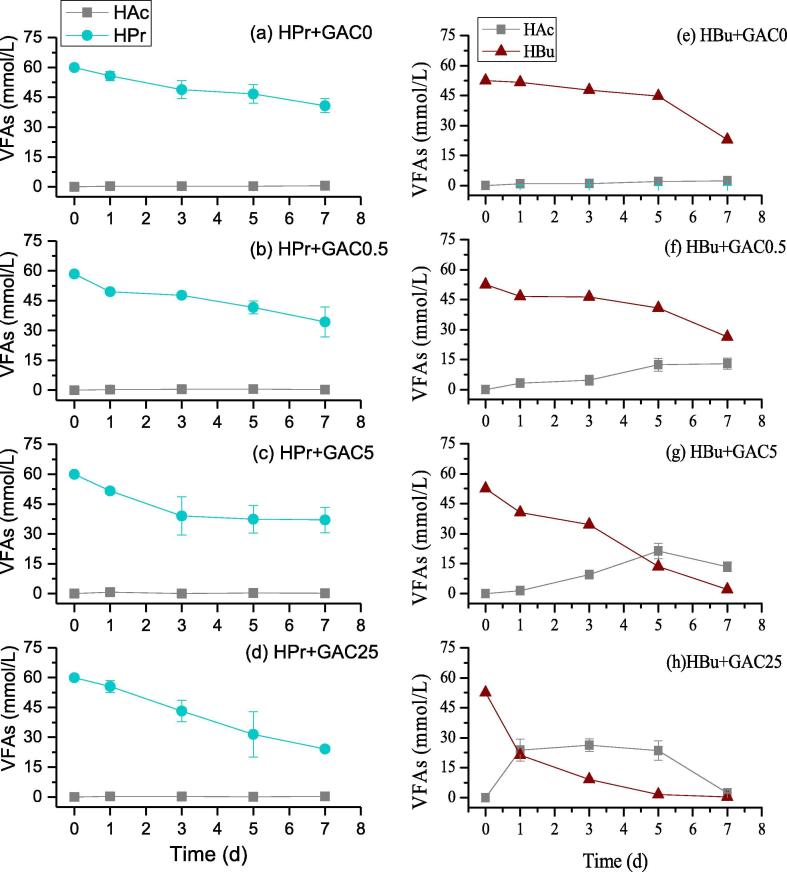
Variations of acetogenic intermediate during the anaerobic degradation of HPr (a–d) and HBu (e–h) at varied GAC dosages.

The dynamics of substrate and intermediate products were presented in Fig. 4. Different accumulation of intermediate product i.e. acetate was found in digesters with different substrate and GAC dosages. Scholten and Conrad (2000) also found the accumulation of acetate in reactor fed with propionate or butyrate as the sole substrate. The syntrophic interaction is essential to overcome the thermodynamic barriers in the anaerobic oxidation of fatty acids. The main pathways involved in the syntrophic degradation of acetate, propionate and butyrate are shown as follows

(3) $${\text{CH}}_{3}{\text{CH}}_{2}{\text{CH}}_{2}{\text{COO}}^{-}+2{\text{H}}_{2}\text{O}\to 2{\text{CH}}_{3}{\text{COO}}^{-}+{\text{H}}^{+}+2{\text{H}}_{2}\;\Delta {\text{G}}^{0\prime }=+48.1\text{kJ}/\text{mol}$$

(4) $${\text{CH}}_{3}{\text{CH}}_{2}{\text{COO}}^{-}+2{\text{H}}_{2}\text{O}\to {\text{CH}}_{3}{\text{COO}}^{-}+{\text{CO}}_{2}+3{\text{H}}_{2}\;\Delta {\text{G}}^{0\prime }=+76.0\text{kJ}/\text{mol}$$

(5) $${\text{CH}}_{3}{\text{COO}}^{-}+{\text{H}}^{+}+2{\text{H}}_{2}\text{O}\to 2{\text{CO}}_{2}+4{\text{H}}_{2}\;\Delta {\text{G}}^{0\prime }=+94.9\text{kJ}/\text{mol}$$

(6) $${\text{CH}}_{3}{\text{COO}}^{-}+{\text{H}}_{2}\text{O}\to {\text{HCO}}_{3}^{-}+{\text{CH}}_{4}\;\Delta {\text{G}}^{0\prime }=-31.0\text{kJ}/\text{mol}$$

(7) $${\text{HCO}}_{3}^{-}+4\text{H}2+{\text{H}}^{+}\to {\text{CH}}_{4}+3{\text{H}}_{2}\text{O}\;\Delta {\text{G}}^{0\prime }=-135.6\text{kJ}/\text{mol}$$

The Gibbs free energy of Eqs. (3), (4), (5) are quite high, which turns to be exergonic reaction only at the low partial pressure of H_2_ or low concentration of acetate (Müller et al., 2010). Thus, the syntrophic degradation requires both hydrogenotrophic and aceticlastic methanogens to consume H_2_ and acetate.

In digesters with propionate as substrate, a slow declination rate of propionate was observed and the accumulation of intermediate product acetate was barely detected. Whereas, acetate was found to be accumulated in digesters with butyrate as substrate (Fig. 4e–h). As the inoculum is the enriched culture mixture of syntrophic acetogenic bacteria and methanogenesis, the detected concentration of acetate was the balance result between syntrophic acetogenesis and methanogenesis. By observing the status of acetate accumulation, results indicate that a slight depression of methanogenesis was occurred in digesters with propionate and butyrate, meanwhile the supplementation of GAC could trigger the acetogenic conversion of propionate and butyrate. The study of Viggi et al. (2014) had evaluated the maximum electron carrier flux occurred in digester with conductive magnetite particles, i.e. DIET which rate is 10^6^ higher than that associated with interspecies H_2_ transfer. Such a scenario could be applied to present study in degrading butyrate and propionate but with a slower rate of electron carrier flux as the conductivity of GAC was smaller than magnetite particles.

In addition, it is noted that the substrate of butyrate tends to be exhausted in GAC25 within 8 days (Fig. 4 h) whereas the methane generation continued to 14 days (Fig. 2f). On one side, apart from acetate other intermediates such as formate and H_2_ might existed in the digesters to contribute for the methane generation. As evidenced by Fig. S1, the remaining TOC concentration was about 211 mg/L when butyrate nearly consumed at Day 7. Nevertheless, the concentration of hydrogen has not been monitored in this study, which warrants further investigations. On the other side, the remaining methane production might be derived from the absorbed substrate on GAC at high dosage. The results of adsorption experiment showed that the adsorption capacity of GAC for HAc, HPr and HBu were 22, 25 and 38 mg/g, respectively.

### 3.4 Characteristics of microbial community

**Fig. 5:**
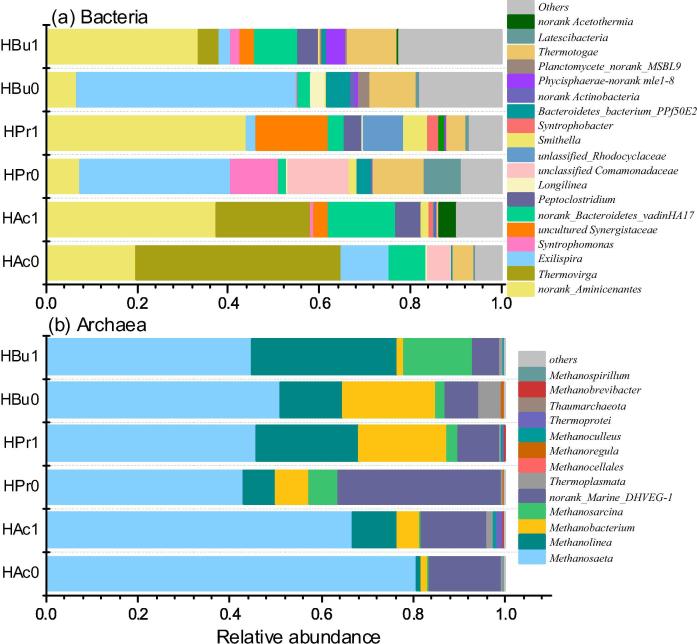
Bacterial (a) and Archaeal (b) community structure at genus level in the anaerobic sludge with 5 g/L GAC and without GAC.

This study compared microbial population enriched with different substrates, i.e. acetate, propionate and butyrate, as well as the effects of GAC supplementation by comparing GAC0 and GAC5. The relative abundance of bacterial and archaeal community at genus level are presented in Fig. 5 and Table S3.

#### 3.4.1 Bacteria

The bacterial community structure changed along with different incubation condition. In reactors fed with acetate, *Aminicenantes* and *Thermovirga* were the predominant bacteria (Fig. 5a). Until now there are only three *Aminicenantes* genomes have been sequenced and no cultured representatives of this lineage. Nevertheless, putative genes for formate dehydrogenase (i.e., hydrogenase-3 and formate hydrogenylase) have been identified in *Aminicenantes* species and could be used to convert formate to hydrogen and carbon dioxide as terminal products of fermentation (Robbins et al., 2016). One OTU clustered to *Aminicenantes* was also reported to use Wood-Ljungdahl pathway in reverse to consume acetate and generate CO_2_ in syntrophic association with a hydrogenotrophic methanogen of the order *Methanomicrobiales* (Gies et al., 2014). As shown in Fig. S2, the function of *Aminicenantes* is closely clustered to Syntrophobacter, which might make contribution to the syntrophic degradation of VFAs.

*Thermovirga* accounted for 45% and 21% of the total OTUs in GAC0 and GAC5 fed with acetate. *Thermovirga* together with *Aminivibrio*, *Acetothermia* are attached to the family of Synergistaceae under the phylum of Synergistetes. Similarly, some identified acetate degrading bacteria belong to the Synergistetes, which is probably syntrophic acetate oxidation coupled with hydrogenotrophic methanogens (Ito et al., 2011). It has found that the identified Synergistetes group had a lower affinity to acetate and a higher acetate utilization rate than *Methanosaeta*-like acetoclastic methanogen (Ito et al., 2011). Thus, *Methanosaeta* and Synergistes group seem to be not competitive, but cooperative for fluctuating concentration of acetate in the anaerobic batch reactor used in this study.

The composition of *Thermovirga* was relatively low in GAC0 fed with propionate and butyrate, whereas a higher proportion of *Exilispira* was detected, which is affiliated to the phylum S*pirochae*te. Bacteria within the *Spirochaetes* are frequently detected in anaerobic digestion systems that treat municipal sludge, livestock wastewater and synthetic organic matters (Lee et al., 2013). The selective enrichment of *Spirochaetes* was reported in reactors accepting fatty acids especially acetate as substrate, suggesting the possible role of *Spirochaetes* in syntrophic acetate oxidation (Lee et al., 2013).

It also found that the relative abundance of *Syntrophomonas* and *Smithella* were higher in propionate reactors, which members are propionate and butyrate oxidizers (Müller et al., 2010, Mcinerney et al., 1981). Liu et al. (2011) had examined the organisms involved in syntrophic oxidation of butyrate in paddy soil using DNA based stable isotope probing, where *Syntrophomonas* spp. together with methanogens *Methanosarcina* and *Methanocella* were the most active. It seems that the syntrophic fatty acids oxidation community was susceptible to the inoculum source.

#### 3.4.2 Archaea

The relative abundance of Archaea in sludge samples ranged from 21% to 61% as seen in Table S3. Comparatively, *Methanosaeta, Methanobacterium, Methanosarcina* and *Methanolinea* are the dominant archaea species in the enriched culture degrading VFAs as shown in Fig. 5b. *Methanosaeta* is a typical acetoclastic methanogen, while *Methanobacterium* and *Methanolinea* belongs to the hydrogenotrophic methanogen species. Methanosarcina produce CH_4_ through three pathways using H_2_/CO_2_, acetate and methylated one-carbon compounds (De Vrieze et al., 2012).

**Fig. 6:**
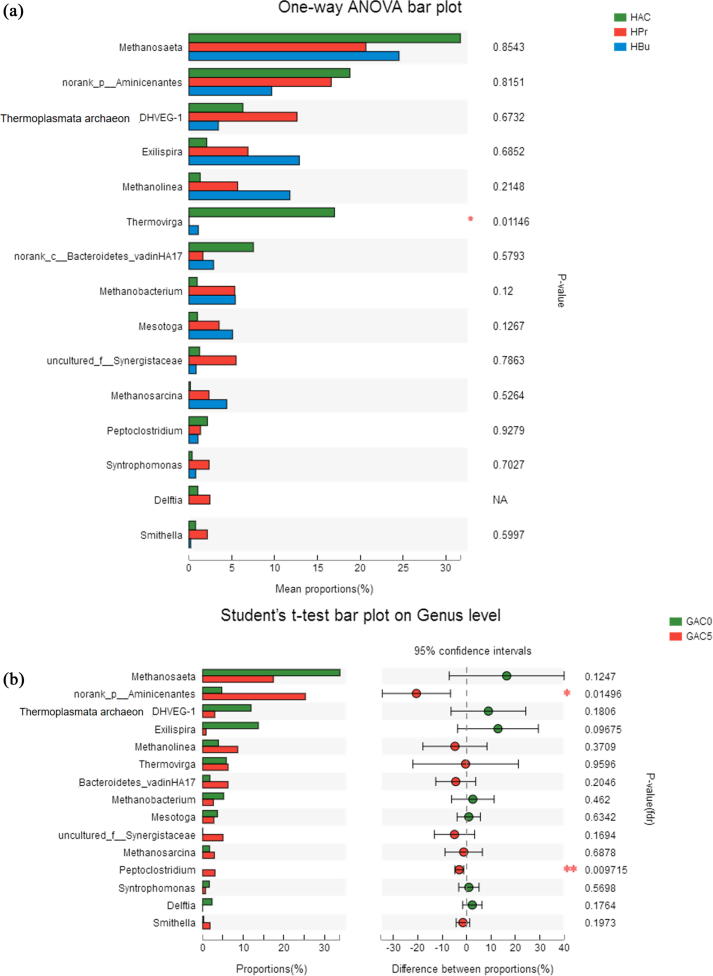

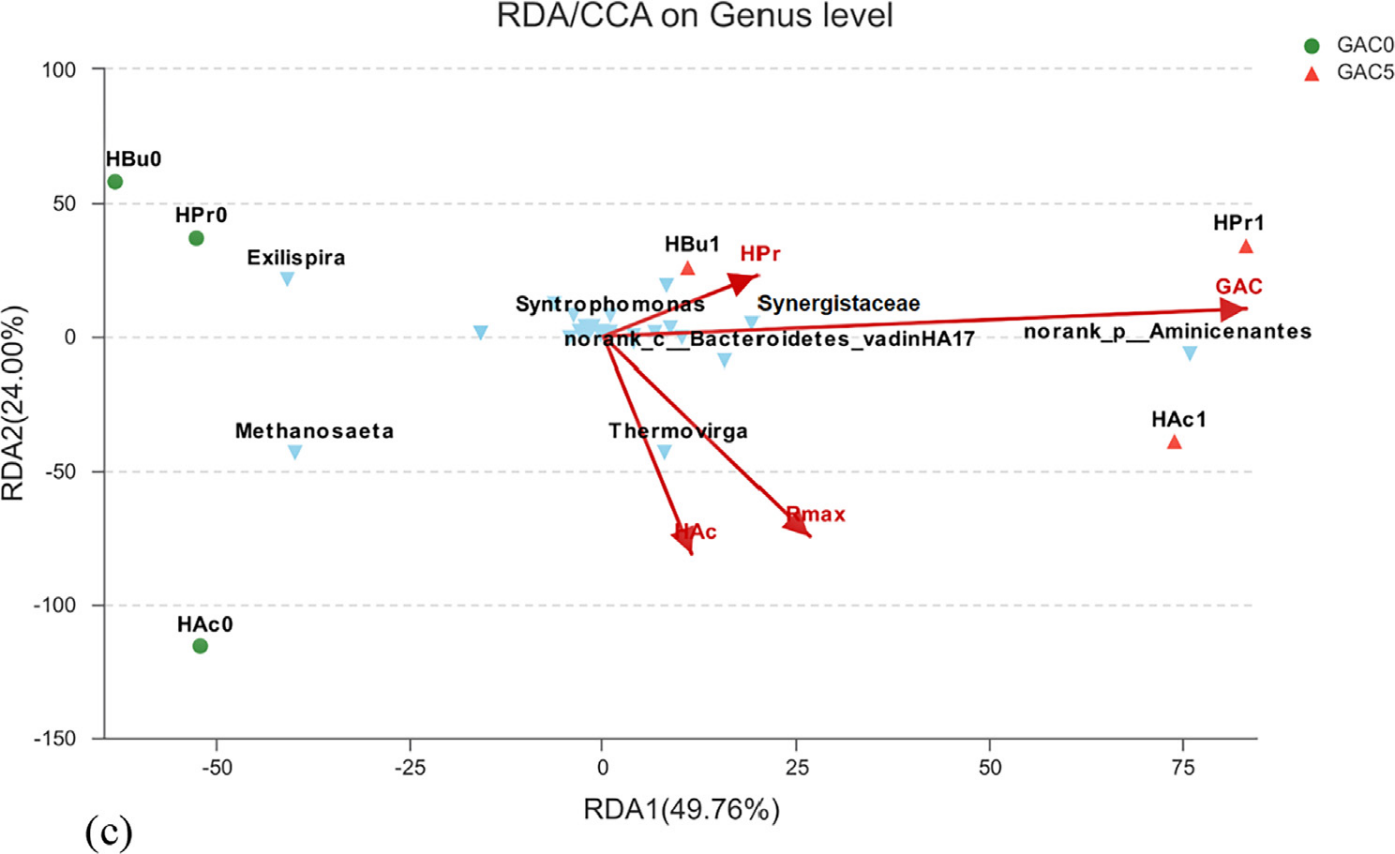
Difference analysis of microbial community on genus level among the groups of HAc, HPr and HBu (a) and between the groups of 0 g/L and 5 g/L GAC (b), and representational difference analysis for the microbial community converging environmental factors of GAC, *R*_max_ and VFAs concentration (c).

On one side, although the selectively enriched archaea varied among reactors with different substrates, *Methanosaeta* predominated in all the reactors (Fig. 5b), and the relative abundance of *Methanosaeta* followed the trend of acetate > butyrate > propionate (Fig. 6a). In acetate-fed reactors, *Methanosaeta* attributed for ∼80.7% of methanogens (HAc0), meanwhile in the propionate- and butyrate-fed reactors, it accounted for 42.8% and 44.8%. Zhao et al. (2016) also reported that *Methanosaeta* species were predominant with either butyrate or propionate as the substrate. The high abundance of *Methanosaeta* could be affiliated to the suitable concentration of acetate (max. 68 mM), and it could also consume electrons derived from the oxidation of propionate or butyrate to acetate.

On the other side, the relative abundance of *Methanosaeta* in the digester with GAC generally decreased as compared to the control without GAC. Similar trend was found for *Methanobacterium*. Whereas the abundance of *Methanosarcina* and *Methanolinea* increased. It has been reported that the presence of *Methanosarcina* could shortly eliminate the negative effect of acidity accumulation and produce methane in high performance (Wang et al., 2017). To data, *Methanosarcina* and *Methanosaeta* are the only methanogens known to participate in DIET by directly receiving electrons to reduce CO_2_ and produce CH_4_ (Rotaru et al., 2014a, Rotaru et al., 2014b). The promoting mechanism of DIET has been well understood in ethanol metabolism with a co-culture of *Geobacter metallireducens* and *Methanosarcina barkeri* (Liu et al., 2012, Rotaru et al., 2014b). In total, the proportion of three known hydrogenotrophic methanogens (i.e. *Methanolinea, Methanobacterium and Methanosarcina*) increased in GAC5 than GAC0. Comparatively, the changes on the Archaeal community of in acetate reactors were less than propionate and butyrate reactors, which was in accordance to the similar reaction kinetics of methane generation with varied GAC dosages.

#### 3.4.3 Difference and correlation analysis of microbial community

The influences of various environmental factors such as GAC supplementation, maximum methane yield rate (*R_max_*) and the concentrations of VFAs on the dynamics of microbial community have been analyzed by the representational difference analysis (RDA) and presented in Fig. 6. Firstly, results indicate that the supplementation of GAC was regarded as the major environmental factor influencing the microbial community. The distributions of HAc1 and HPr1 and HBu1 were in the same direction with GAC, whereas HAc0, HPr0 and HBu0 were located on the contrary coordinate. Furthermore, the abundance of syntrophic bacteria such as *Aminicenantes*, *Thermovirga, Synergistaceae* and *Syntrophomonas* were also clustered with the same direction of GAC, whereas *Methanosaeta* was on the contrary coordinate, which is in accordance to the above observation. To data, only *Methanosarcina* and *Methanosaeta* are known methanogens to participate in DIET by directly receiving electrons to reduce CO_2_ and produce CH_4_ (Rotaru et al., 2014a).

*Geobacter* is also known as one important bacterial genus to participate in DIET, which accounted for ca. 20% of the community attached to biochar (Zhao et al., 2016) or GAC (Lee et al., 2016). Lee et al. (2016) found the enrichment of exoelectrogens e.g. *Geobacter* and hydrogenotrophic methanogens (e.g. *Methanospirillum* and *Methanolinea*) from the biomass attached to GAC. However, in this study, the OTUs clustered to *Geobacter* detected in sludge with GAC was quite low (< 1% of total bacteria). Similar result has been previously reported (Xu et al., 2015, Dang et al., 2017, Barua and Dhar, 2017). Thus, it’s a possibility that other organisms rather than *Geobacter* may also participate in DIET with methanogens as suggested (Kato et al., 2012).

Last but not the least, the relative concentration of syntrophic oxidation bacteria and methanogens to the substrate should also be carefully attention (Ferguson et al., 2016). As shown in Fig. 4e and f, the accumulation of acetate was relatively low when GAC concentration was very low, whereas when GAC concentration increased, the conversion rate of butyrate into acetate was accelerated and the accumulation of acetate accordingly elevated. It seems that with the low GAC dosage, microorganism enriched on the GAC surface and associated DIET could eliminate the resistance to acetogenesis, and the bottleneck turns to be the methanogenesis. However, this deduction requires further demonstration as the intermediate products are not fully identified in present study.

## 4 Conclusion

This study has demonstrated that the supplementation of appropriate GAC dosage could accelerate the syntrophic degradation of propionate and butyrate efficiently under heavy organic load. Specifically, the degradation rates of propionate and butyrate were sharply increased by 1.5 and 4.2 times at 5 g/L of GAC as compared to the control (GAC0), nevertheless minor increment was found for *R*_max_ when further increasing GAC dosage to 25 g/L. Therefore, the lower dosage of GAC is recommended to use in anaerobic digester, and economics of this approach for improving digester performance would be favorable. GAC benefits the enrichment of syntrophic oxidation bacteria but need a period of cultivation, thus it is suggested to retain GAC within the continuous feeding digester.
