# Supplementary material for: Differentiated stimulating effects of activated carbon on methanogenic degradation of acetate, propionate and butyrate
Source: Waste Manag. 2018 Jun;76:394–403. doi: 10.1016/j.wasman.2018.03.037 (PMC5980998; doi:10.1016/j.wasman.2018.03.037)
Supplement: Supplementary data 1 [file mmc1.docx]

**Supporting information**

**For**

**Differentiated stimulating effects of activated carbon on syntrophic degradation of acetate, propionate and butyrate**

**Suyun Xu*, Runqi Han, Yucheng Zhang, Chuanqiu He, Hongbo Liu****

*Department of Environment & Low-Carbon Science, School of Environment and Architecture, University of Shanghai for Science and Technology, Shanghai 200093, China*

**Corresponding author: Email:* [xusy@usst.edu.cn](mailto:xusy@usst.edu.cn), *Tel./fax: 8621 5527 5979*

***Common corresponding author: Email:* [*Liuhb@usst.edu.cn*](mailto:Liuhb@usst.edu.cn)*,*

Total number of pages: 7

Total number of Figures: 2

Total number of Table: 3

**Table** S1**.** Comparison of the dosages and stimulating effects of carbon materials on syntrophic degradation of VFAs

| Additives dosage | Substrate | Effect | Enriched microbial community |  |
| --- | --- | --- | --- | --- |
| 50 g/L GAC | OFMSW | significantly enhanced COD removal efficiency and methane production | *Methanosaeta* was the dominant archaea;  *Geobacter* not enriched; *Sporanaerobacter* likely to participate in DIET with methanogens | (Dang et al., 2017) |
| 1 g/L GAC | Acetate | 1.8-fold higher methane yield | *Geobacter* and hydrogenotrophic methanogens | (Lee et al., 2016) |
| 5 g/L biochar | Ethanol/butyrate  Ethanol/propionate | Butyrate: 25% higher methane yield  Propionate: 16% higher methane yield | *Geobacter and Methanosaeta* enriched on the surface of biochar | (Zhao et al., 2016) |
| 0.005 g/L biochar | Molasses | - | Increased abundance of *Methanosaetaceae* | (De Vrieze et al., 2016) |
| 2.5 g/L biochar  & carbon cloth | Ethanol | Syntrophic metabolism declined when biochar or carbon closth were removed from reactor | - | (Zhao et al., 2015) |
| 10 g/L biochar | Glucose | Increased methane production rate by 70.6% at 6 g/L glucose loading | *Methanosarcina* located in the tightly-bound fractions on the biochar surface, and *Methanosaeta* was enriched in the loosely-bound fractions | (Luo et al., 2015) |
| 20 g/L biochar | N-rich substrates, | 32% of increment on methane yield | - | (Mumme et al., 2014) |
| 25 g/L GAC | Ethanol/ fumarate | 40% faster of ethanol metabolism | Co-cultures of *G. metallireducens and Methanosarcina barkeri* | (Liu et al., 2012) |

**Table** S2. Calculated results using the modified Gompertz equation for methanogeneis with varied GAC dosages

|  | GAC  Dosage | λ (d) | *R_max_*  (mmol-CH_4_/  mmol-C_added_/d) | V_max_ ^a^  (mmol-CH_4_/L/d) | *P_CH4_*  (mmol-CH_4_/  mmol-C_added_) | *R^2^* |
| --- | --- | --- | --- | --- | --- | --- |
| *1 g/L HAc* | 0 g/L | 0.86±0.22 | 0.16±0.02 | 6.36 | 0.45 | 0.98 |
|  | 0.5 g/L | 0.60±0.14 | 0.21±0.03 | 8.35 | 0.49 | 0.99 |
|  | 5 g/L | 0.53±0.09 | 0.19±0.02 | 7.55 | 0.48 | 0.99 |
|  | 25 g/L | 0.16±0.39 | 0.14±0.03 | 4.37 | 0.46 | 0.99 |
| *5 g/L HAc* | 0 g/L | 0.29±0.11 | 0.26±0.03 | 24.47 | 0.44 | 0.99 |
|  | 0.5 g/L | 0.29±0.11 | 0.25±0.03 | 24.47 | 0.44 | 0.99 |
|  | 5 g/L | 0.40±0.02 | 0.34±0.01 | 33.75 | 0.46 | 0.99 |
|  | 25 g/L | 0.36±0.06 | 0.33±0.02 | 30.38 | 0.45 | 0.99 |
| *1 g/L HPr* | 0 g/L | 0.52±0.11 | 0.13±0.01 | 3.19 | 0.36 | 0.99 |
|  | 0.5 g/L | 0.64±0.10 | 0.13±0.01 | 3.19 | 0.36 | 0.99 |
|  | 5 g/L | 0.62±0.11 | 0.12±0.01 | 2.95 | 0.36 | 0.99 |
|  | 25 g/L | 0.70±0.09 | 0.081±0.011 | 1.99 | 0.35 | 0.99 |
| *5 g/L HPr* | 0 g/L | 4.17±0.0.28 | 0.005±0.001 | 0.25 | 0.018 | 0.92 |
|  | 0.5 g/L | 3.41±0.21 | 0.007±0.001 | 0.35 | 0.026 | 0.97 |
|  | 5 g/L | 1.18±0.35 | 0.038±0.001 | 1.88 | 0.179 | 0.97 |
|  | 25 g/L | 0.94±0.34 | 0.032±0.003 | 1.59 | 0.170 | 0.97 |
| *1 g/L HBu* | 0 g/L | 1.01±0.14 | 0.16±0.02 | 2.56 | 0.47 | 0.99 |
|  | 0.5 g/L | 0.95±0.17 | 0.17±0.02 | 2.72 | 0.48 | 0.99 |
|  | 5 g/L | 0.73±0.16 | 0.17±0.02 | 2.72 | 0.49 | 0.99 |
|  | 25 g/L | 1.04±0.22 | 0.15±0.02 | 2.40 | 0.48 | 0.99 |
| *5 g/L HBu* | 0 g/L | 12.67±0.071 | 0.015±0.001 | 0.49 | 0.06 | 0.99 |
|  | 0.5 g/L | 10.94±0.076 | 0.015±0.001 | 0.49 | 0.07 | 0.99 |
|  | 5 g/L | 5.15±0.200 | 0.054±0.002 | 1.77 | 0.49 | 0.99 |
|  | 25 g/L | 7.83±0.411 | 0.098±0.012 | 3.22 | 0.64 | 0.96 |

**^a^** V_max_ is the maxium volumetric CH_4_ production rate

**Table S3**. The proportion of Archaea and Bacteria in sludge samples

|  | HAc0 | HAc1 | HPr0 | HPr1 | HBu0 | HBu1 |
| --- | --- | --- | --- | --- | --- | --- |
| Archaea | 20963 | 7841 | 19194 | 13697 | 17285 | 17625 |
| Bacteria | 13288 | 29628 | 11122 | 30348 | 17865 | 15260 |
| Total OTUs | 34251 | 37469 | 30316 | 44045 | 35150 | 32885 |
| Archaea of total OTUs, % | 61.2 | 20.9 | 63.3 | 31.1 | 49.2 | 53.6 |
| Bacteria of total OTUs, % | 38.8 | 79.1 | 36.7 | 68.9 | 50.8 | 46.4 |
| Methanosaeta of Archaea % | 80.7 | 66.8 | 42.8 | 45.9 | 51.1 | 44.8 |
| Thermovirga of Bacteria % | 44.9 | 21.0 | 0 | 0.06 | 0.1 | 4.6 |

**Fig.** S1. Variations of TOC concentration during digestion with initial VFA concentration of 5 g/L.


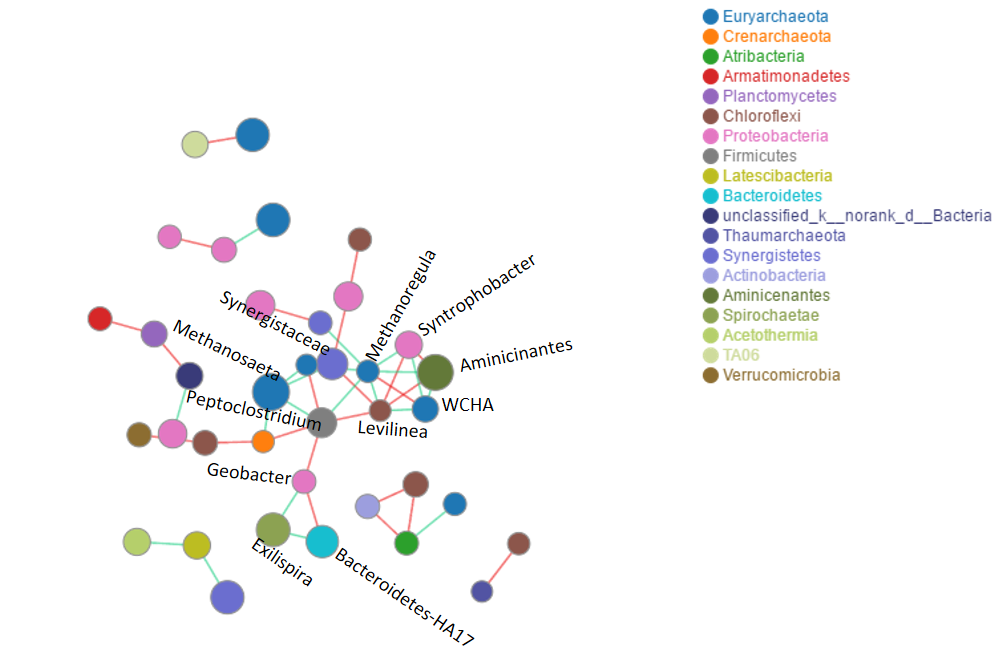


**Fig.** S2 Network analysis on genus level

High throughout sequencing data sets are also highly amenable to network analysis which can offer novel insights into community composition, taxonomic interactions, and ecological niche associations in complex microbial communities (Oakley et al., 2013).

**References**

Dang, Y., D. Sun, T. L. Woodard, L. Y. Wang, K. P. Nevin, D. E. Holmes, Stimulation of the anaerobic digestion of the dry organic fraction of municipal solid waste (OFMSW) with carbon-based conductive materials. *Bioresour. Technol.* **238**, 2017, 30-38.

De Vrieze, J., A. Devooght, D. Walraedt, N. Boon, Enrichment of Methanosaetaceae on carbon felt and biochar during anaerobic digestion of a potassium-rich molasses stream. *Appl. Microbiol. Biotechnol.* **100**, 2016, 5177-5187.

Lee, J.-Y., S.-H. Lee, H.-D. Park, Enrichment of specific electro-active microorganisms and enhancement of methane production by adding granular activated carbon in anaerobic reactors. *Bioresour. Technol.* **205**, 2016, 205-212.

Liu, F., A.-E. Rotaru, P. M. Shrestha, N. S. Malvankar, K. P. Nevin, D. R. Lovley, Promoting direct interspecies electron transfer with activated carbon. *Energ. Environ. Sci.* **5**, 2012, 8982.

Luo, C., F. Lu, L. Shao, P. He, Application of eco-compatible biochar in anaerobic digestion to relieve acid stress and promote the selective colonization of functional microbes. *Water Res.* **68**, 2015, 710-718.

Mumme, J., F. Srocke, K. Heeg, M. Werner, Use of biochars in anaerobic digestion. *Bioresour. Technol.* **164**, 2014, 189-197.

Oakley B B, Morales C A, Line J, Berrang M, Meinersmann R, Tillman G, Wise M, Siragusa G, Hiett K, Seal B, The poultry-associated microbiome: network analysis and farm-to-fork characterizations. *Plos One* **8**, 2013, e57190.

Zhao, Z., Y. Zhang, D. E. Holmes, D. Yan, T. L. Woodard, K. P. Nevin, D. R. Lovley, Potential enhancement of direct interspecies electron transfer for syntrophic metabolism of propionate and butyrate with biochar in up-flow anaerobic sludge blanket reactors. *Bioresour. Technol.* **209**, 2016, 148-156.

Zhao, Z., Y. Zhang, T. Woodard, K. Nevin, D. Lovley, Enhancing syntrophic metabolism in up-flow anaerobic sludge blanket reactors with conductive carbon materials. *Bioresour. Technol.* **191**, 2015,140-145.
